# Race, Diet, and Cardiometabolic Risk in US Young Adults: A Biosocial Framework Integrating Biological Mechanisms and Structural Inequities Toward Precision Nutrition

**DOI:** 10.1007/s13668-026-00804-x

**Published:** 2026-10-12

**Authors:** Onyedika G. Ani

**Affiliations:** Department of Nutrition and Exercise Physiology, University of Missouri, Columbia, Missouri USA

**Keywords:** Cardiometabolic disease, Race disparities, Functional foods, Precision nutrition, Gut microbiome, Multi-omics

## Abstract

**Purpose of Review:**

Cardiometabolic disease disproportionately affects Black populations in the US, yet dietary interventions have not reduced these disparities. This review examines race as a biosocial construct integrating ancestry-related biology and structural exposures to understand cardiometabolic risk and dietary response in precision nutrition

**Recent Findings:**

Evidence suggests racial differences in glucoregulatory physiology, metabolically relevant allele frequencies, stress-related epigenetic regulation, gut microbiome composition, and metabolomic profiles. Functional foods, including fruits, vegetables, and nuts, target pathways linked to these phenotypes. However, most intervention studies involve older, predominantly White populations, limiting understanding of race-specific responses.

**Summary:**

The review proposes the “cumulative racial biological phenotype” framework, where ancestry-related biology and structural exposures jointly influence cardiometabolic risk. It advances the greater plasticity hypothesis, suggesting greater baseline biological perturbation may predict larger dietary responses. Testing these concepts requires race-stratified, multi-omics dietary intervention trials in young adults using culturally and structurally responsive designs to improve the effectiveness and equity of precision nutrition.

**Supplementary Information:**

The online version contains supplementary material available at https://doi.org/10.1007/s13668-026-00804-x.

## Introduction

Cardiometabolic diseases (CMD) remain a leading cause of morbidity and premature mortality in the US, with disproportionate burden concentrated in non-Hispanic Black populations. Black adults experience type 2 diabetes (T2D) at 1.75 times the rate of non-Hispanic White adults (21.1% vs. 12.0%), hypertension at 56.9% vs. 43.5%, and obesity at 49.9% vs. 41.4%, based on NHANES 2017-March 2020 surveillance [1]. These disparities are not confined to older adults or lower socioeconomic strata; they emerge in young adulthood and widen across the life course despite overall population-level improvements in cardiovascular mortality [2]. The failure to appropriately recognize these disparities and proffer targeted intervention has perpetuated the increased CMD prevalence among the Black population.

Dietary recommendations draw on evidence from trials whose participants do not always reflect the full diversity of the populations to which the recommendations are applied. For example, the landmark PREDIMED trial, which established the Mediterranean diet as cardioprotective, enrolled 7,447 participants with a mean age of 67 years, predominantly of European descent [3]. Similarly, the well-known DASH trial included a substantial proportion of Black participants, yet was not designed or powered to rigorously assess race-specific differences in dietary response [4]. These limitations warrant more inclusive intervention studies and careful assessment of how well trial findings apply to underrepresented populations, especially the Blacks.

**Fig. 1:**
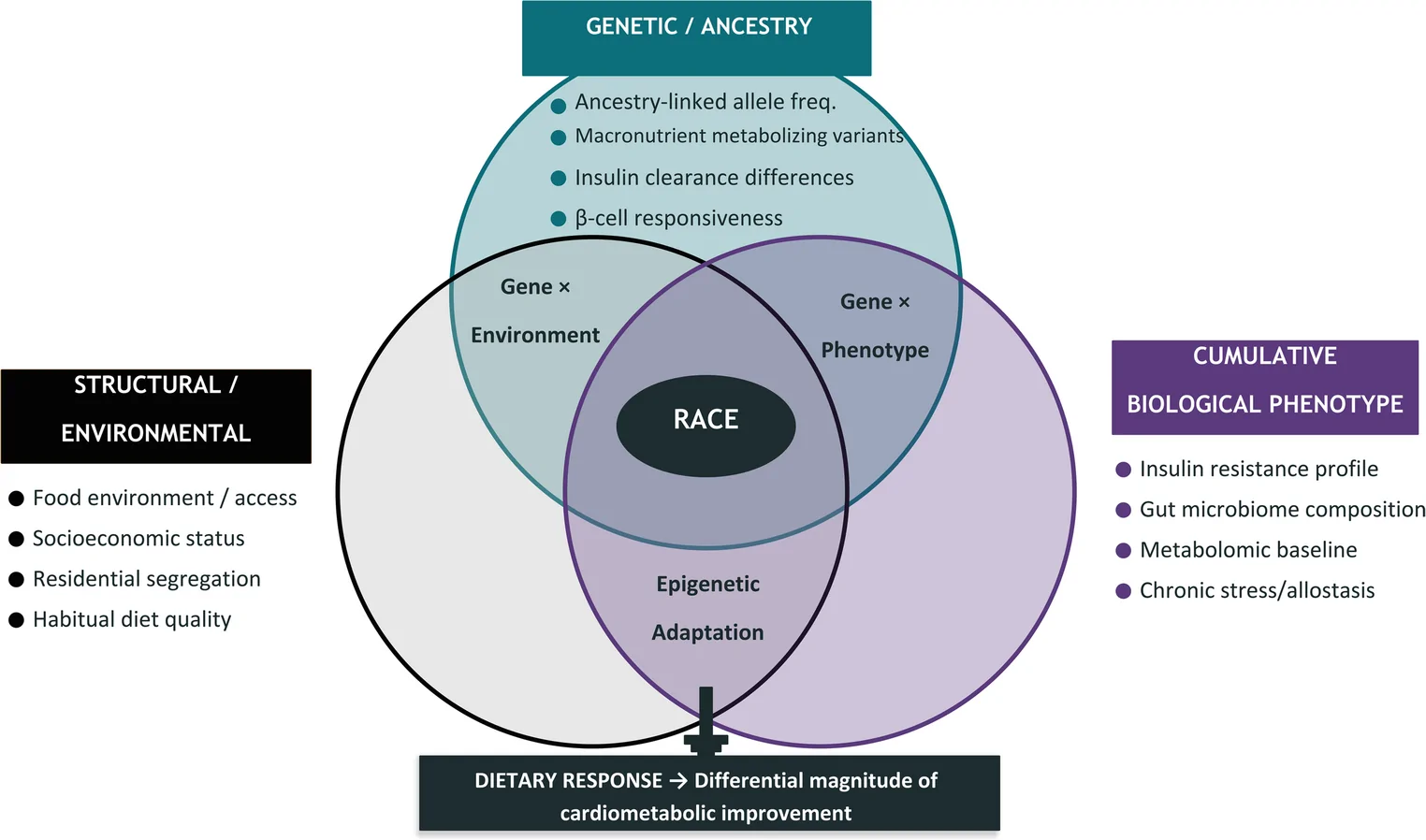
Race as a biosocial construct encompasses different domains that interact to drive differential cardiometabolic risk and dietary response

This review presents a race-centered conceptual framework that positions race as the primary organizing variable in cardiometabolic nutrition research instead of merely a demographic covariate. Self-identified race is a social classification that can help identify inequities in exposure and health outcomes, but it does not identify an individual’s genetic ancestry or biological characteristics. This review considers genetic ancestry, measured glucoregulatory and microbiome characteristics, and structural exposures, including neighborhood food access, residential segregation, and chronic psychosocial stress, as distinct factors that may interact to shape cardiometabolic risk (Fig. 1). The genetic dimensions continuously interact with structural exposures, influencing biological phenotype through epigenetic modifications, gut microbiome dysbiosis, and neuroendocrine programming. These biological variations affect the extent to which structural exposures lead to cardiometabolic risks. Together, they form the cumulative racial biological phenotype, the modifiability of which through diet remains the key scientific question of this review.

This review specifically targets young adults during the developmental window when cardiometabolic risk factors develop. During this stage, preventive dietary intervention offers the most biological advantage. The review identifies four key gaps: paucity of race-stratified dietary trials, microbiome response data, metabolomic evidence, and integrated multi-omics interventions in diverse young adults. Collectively, the evidence supports the need to consider race as a modifier in dietary intervention improving CMD risks in young adults.

### Methods

This review synthesized evidence from observational human studies, randomized controlled trials, and mechanistic experimental studies on diet, cardiometabolic risk, and biological pathways relevant to metabolic regulation. Literature was identified through searches of PubMed, Scopus, and Web of Science up to January 2026 using combinations of keywords including “cardiometabolic disease,” “diet quality,” “race,” “black,” “white,” “insulin sensitivity,” “gut microbiome,” “metabolomics,” and “precision nutrition.” Additional articles were identified through reference list screening of relevant reviews and primary studies. Priority was given to studies with direct mechanistic relevance, multi-omics integration, or racially diverse cohorts. Given the narrative nature of this review, formal systematic inclusion criteria were not applied; however, emphasis was placed on consistency of findings across multiple lines of evidence.

## Racial Cardiometabolic Disparities: Epidemiology and the Young Adult Case

### Prevalence and Early Onset

National surveillance data consistently show significant racial disparities in cardiometabolic conditions. Non-Hispanic Black adults have the highest age-adjusted rates of obesity, hypertension, and type 2 diabetes among major racial groups [1] (Table 1). Notably, these disparities emerge early in life. Among those aged 20–39, obesity prevalence in Black adults already surpasses 40%, and differences in blood pressure regulation and insulin sensitivity are evident by the third decade of life [1]. The early onset of CMD risks highlights the need for preventive interventions to hinder further deterioration across the lifespan.

Obesity, a complex metabolic disorder characterized by excess adiposity resulting from sustained positive energy balance, is driven by interacting behavioral, environmental, and genetic factors, including poor diet quality, physical inactivity, and inherited susceptibility [5]. Its progression is accompanied by a constellation of metabolic syndrome (MetS), including dyslipidemia, insulin resistance, and hypertension, that collectively contribute to the development of CMD [5] (Supplementary Fig. 1). However, framing racial disparities solely in terms of body mass index (BMI), on which obesity is measured, may underestimate their biological significance. Black adults show distinct patterns of fat distribution, including differences in visceral and ectopic fat deposition relative to BMI [6, 7], which may contribute to disproportionate metabolic risk. These differences are associated with greater insulin resistance and altered adipokine profiles [8], suggesting that equal BMI values do not reflect the same metabolic burden across racial groups.

Behavioral risk factors further contextualize these racial disparities. NHANES 2011–2018 data (*n* = 10,405; ages 18–44) indicate that non-Hispanic Black young adults have the highest prevalence of poor diet (56.0%), inadequate sleep (49.2%), and physical inactivity (29.6%) compared to White adults (Table 1), but do not show the highest rates of smoking or excessive alcohol use [9]. Though the pattern supports the high prevalence of obesity, it does suggest that the increased cardiometabolic risk observed in this population is not solely due to all adverse health behaviors. Rather, deficits in diet quality, sleep, and physical activity, key determinants of gut microbiome composition, insulin sensitivity, and metabolic inflammation, provide a coherent biological framework for understanding early risk accumulation. Together, these findings underscore the importance of targeted, mechanism-informed nutrition interventions in young adulthood, particularly among populations disproportionately affected by early cardiometabolic risk.

**Table 1:** Age-adjusted prevalence (%) of major cardiometabolic risk factors and selected lifestyle determinants by race in the US

Race/ethnicity	Obesity (%)	Hypertension (%)	Type 2 diabetes (%)	Poor diet quality (%)*	Inappropriate sleep (%)*	Inadequate physical activity (%)*
Non-Hispanic Black	49.9	56.9	21.1	56.0*	49.2*	29.6*
Hispanic	45.6	43.5	18.1	46.1	37.0	30.5
Non-Hispanic White	41.4	44.6	12.0	50.4*	33.1*	22.1*
Non-Hispanic Asian	16.1	42.7	18.8	33.5	28.2	33.1

Prevalence estimates for obesity, hypertension, and type 2 diabetes are drawn from NHANES 2017-March 2020. Lifestyle risk factor estimates are drawn from NHANES 2011–2018 among adults aged 18–44 years. *Statistically significant difference versus at least one other racial group (*p* < 0.05). Adapted from [9].

### Young Adulthood as the Priority Intervention Window

Young adulthood is a crucial period for preventing CMD, as this stage occurs before metabolic dysfunction usually becomes evident in midlife. The concept of metabolically healthy obesity (MHO) is particularly relevant during this time, when individuals may have obesity without obvious metabolic problems [10]. However, evidence from longitudinal studies indicates that MHO is often temporary, with about 30–50% of people progressing to metabolically unhealthy obesity within ten years [11, 12]. This transition marks the gradual decline in metabolic homeostasis over the lifespan, which may be influenced by reduced adipose tissue buffering capacity, ectopic lipid accumulation, gut microbiome dysbiosis, chronic low-grade inflammation, worsening insulin resistance, and β-cell stress and eventual dysfunction (Supplementary Fig. 2). This progressive deterioration mirrors the events at the onset and during the progression of obesity, supporting its chronicity.

A conceptual progression as illustrated in Supplementary Fig. 3, in which obesity leads to adipocyte hypertrophy, resulting in dyslipidemia and ectopic lipid deposition. These processes are closely associated with adipose tissue dysfunction and metabolic complications [13, 14]. Excessive lipid accumulation impairs insulin signaling, leading to insulin resistance, as evidenced by hypertrophic adipocytes overloaded with lipids, even in the absence of overt inflammation [15]. As adipose tissue continues to expand, it becomes a source of chronic inflammatory mediators, including cytokines such as TNF-α and IL-6, which contribute to systemic inflammation [13, 16]. Pancreatic β-cell insulin secretion increases to maintain normoglycemia despite impaired insulin action, leading to hyperinsulinemia [17, 18]. Notably, these processes often begin well before clinical signs of MetS emerge, suggesting that the biological pathway to CMD is already established in early adulthood but fully manifests in older adulthood [2]. Addressing these issues at this stage, before or during metabolic instability, greatly increases the likelihood of reducing CMD risk compared with attempting to reverse these conditions later in life.

Prospective cohort studies also show that modest elevations in cardiometabolic risk factors, such as blood pressure, glucose, Low-density Lipoprotein Cholesterol (LDL-C), and BMI, during young adulthood are strongly associated with future cardiovascular events [19, 20]. The total exposure over time appears to be as important as the absolute level of any single marker, indicating that early, subclinical risk in adulthood can significantly affect long-term disease outcomes. Several reinforcing processes make this life stage especially important. For instance, adiposity patterns formed in early adulthood tend to last [21], while insulin resistance can become self-perpetuating through interactions with inflammation and weight gain [22]. Dietary habits and physical activity patterns become ingrained, and the gut microbiome may settle into states that mirror long-term dietary exposures. At the same time, chronic psychosocial stress and structural disadvantages may affect neuroendocrine and epigenetic pathways that further entrench cardiometabolic risk.

These dynamics are especially evident in the context of racial disparities. Black young adults are disproportionately likely to follow trajectories marked by earlier and faster metabolic decline, yet they remain underrepresented in dietary intervention research aimed at preventing this shift. This gap signifies both a crucial scientific shortfall and a moral obligation for equity. Focusing on young adulthood, especially among high-risk groups, offers a strategic chance to intervene when biological and public health impacts are greatest.

### Ancestry-Linked Genetic Architecture

Race is not a purely genetic category, as within-group genetic variation surpasses differences between groups. However, population-level differences in allele frequencies of key functional variants are both prominent and nutritionally significant. The well-studied CD36 variant -rs3211938, which decreases CD36 fatty acid translocase expression by about 50% in heterozygous carriers, has an allele frequency of approximately 25% in West African-ancestry populations but is below 1% in European-ancestry populations [23]. The reported associations of rs3211938 with fatty acid metabolism and cardiometabolic outcomes [24] raise a testable question: does dietary fat quality have different effects in carriers and noncarriers? Although allele frequencies differ between sampled ancestry populations [23], self-identified race cannot establish an individual’s carrier status or predict dietary response. Whether replacing saturated fats with foods rich in monounsaturated and polyunsaturated fatty acids provides greater benefit to carriers requires direct testing.

Similarly, the FADS1/FADS2 locus, which encodes delta-5 and delta-6 desaturase enzymes, presents an equally important but dual-edged gene-diet interaction. The high-activity haplotype, associated with better efficiency in converting linoleic acid (LA) to arachidonic acid (AA) and alpha-linolenic acid (ALA) to eicosapentaenoic acid (EPA)/docosahexaenoic acid (DHA), is found in about 80% of individuals with African ancestry compared to roughly 45% of those with European ancestry [25]. The metabolic effects of FADS1/FADS2 variation may depend on both genotype and dietary fatty acid intake. Whether increasing ALA or providing preformed EPA and DHA produces different lipidomic responses in carriers of particular haplotypes remains a testable question. Self-identified race should not be used to infer haplotype or fatty acid intake. This presents a gene-diet interaction that can be directly tested using measured genotype, dietary intake, and lipidomic outcomes.

Additional variants with differential allele frequencies, including ENaC sodium channel variants conferring greater dietary sodium sensitivity, APOL1 G1/G2 risk alleles associated with kidney disease susceptibility amplified by obesity, and TCF7L2 beta-cell function variants, collectively define a genetic architecture in which the metabolic consequences of dietary exposures are not equivalent across populations (Table 2). This is not biological determinism; these are gene-diet interactions, and their consequences are modifiable by dietary quality.

**Table 2:** Key ancestry-linked genetic variants relevant to cardiometabolic risk and dietary response in African-ancestry populations

Gene / variant	Biological function	Allele freq. (African ancestry)	Allele freq. (European ancestry)	Cardiometabolic relevance	Reference
CD36 Pro90Ser (rs3211938)	Fatty acid translocase; myocyte lipid uptake	~ 25%	< 1%	Dilated cardiomyopathy; impaired FA oxidation; ectopic lipid deposition	[23, 24]
FADS1/2 high-activity haplotype	Delta-5/6 desaturase; LA→AA, ALA→EPA/DHA conversion	~ 80%	~ 45%	Greater AA synthesis; amplified inflammatory eicosanoid production on high-LA diets	[25]
APOL1 G1/G2 risk variants	Apolipoprotein L1; innate immunity; renal podocyte function	~ 13–20% (G1); ~4–5% (G2)	< 1%	CKD, FSGS; risk amplified by obesity and insulin resistance	[26]
TCF7L2 rs7903146 T allele	Transcription factor; beta-cell function; GLP-1 signaling	~ 30%	~ 28%	T2D risk; attenuated effect in African-ancestry vs. European-ancestry samples	[27]
ENaC/SCNN1 variants (sodium channel)	Renal sodium reabsorption; blood pressure regulation	Higher frequency	Lower frequency	Salt-sensitive hypertension; dietary sodium sensitivity in Black adults	[28]

Allele frequencies are approximate population averages from published genomic studies and GWAS data. *FA *= fatty acid, *LA* = linoleic acid *AA* = arachidonic acid, *ALA* = alpha-linolenic acid, *EPA* = eicosapentaenoic acid, *CKD* = chronic kidney disease, *FSGS* = focal segmental glomerulosclerosis, *T2D* = type 2 diabetes mellitus, *ENaC* = epithelial sodium channel

### Epigenetic Adaptation and Cumulative Biological Disadvantage

The epigenome, the layer of gene regulation shaped by DNA methylation, histone modification, and non-coding RNA expression, provides the molecular mechanism through which structural exposures are translated into biological phenotype [29]. Chronic dietary disadvantage depletes the methyl-donor pool (folate, choline, betaine) needed for protective DNA methylation, thereby promoting hypomethylation at inflammatory gene promoters and hypermethylation at insulin-signaling gene loci [30]. Dietary fiber-driven butyrate production inhibits histone deacetylase (HDAC) enzymes, promoting chromatin openness at genes regulating mitochondrial biogenesis and anti-inflammatory macrophage polarization [31]. These mechanisms could be exploited by dietary quality improvement to reverse accumulated epigenetic dysregulation.

The hypothalamic-pituitary-adrenal (HPA) axis, which governs the cortisol stress response, is a well-established target of epigenetic programming by early-life and chronic psychosocial stress. Chronic psychosocial stress from racial discrimination programs the HPA axis through NR3C1 methylation, promoting visceral adiposity and systemic inflammation through sustained cortisol hypersecretion [32]. Likewise, epigenetic modifications of the corticotropin-releasing hormone (CRH) and FKBP5 genes, key regulators of glucocorticoid receptor sensitivity, are also associated with chronic stress exposure and result in persistent alterations in HPA axis reactivity into adulthood [33]. Exposure to discrimination and other chronic stressors is unevenly distributed across race and may contribute to differential cardiometabolic risk through stress-related biological pathways. Epigenetic regulation is one possible pathway, but the extent to which specific dietary interventions alter these effects in humans remains uncertain. Future studies should measure stress exposures, diet, relevant epigenetic markers, and cardiometabolic outcomes directly rather than infer an individual’s epigenetic profile or likely dietary benefit from self-identified race.

### Glucoregulatory Phenotype

Effective regulation of glucose homeostasis is central to maintaining metabolic health and preventing the progression toward CMD. Glucoregulatory control involves a coordinated set of physiological processes, including pancreatic insulin secretion [34], hepatic glucose production and lipid utilization [35], intestinal glucose absorption [36], and peripheral glucose uptake [37], that collectively maintain glucose within a narrow physiological range. Disruptions to this system contribute to the development of insulin resistance, impaired glucose tolerance, T2D, and downstream cardiometabolic complications [38]. This suggests that interventions aimed at restoring glucoregulatory homeostasis, chiefly via improved insulin sensitivity, may reduce the risk of CMD.

Insulin sensitivity, a key determinant of metabolic health, reflects the responsiveness of peripheral tissues, primarily skeletal muscle, adipose tissue, and liver, to circulating insulin [39, 40]. Reduced insulin sensitivity leads to compensatory hyperinsulinemia and, eventually, beta-cell dysfunction, thereby accelerating the onset of T2D [41]. Multiple factors influence insulin sensitivity, including genetic predisposition, adiposity, inflammation, diet quality, and gut microbial composition [42, 43]. Some studies comparing participants classified as Black and White have reported differences in postprandial insulin concentrations, insulin clearance, or beta-cell responses [44]. These are group-level observations in the populations studied, with substantial variation within each group; they do not define a glucoregulatory phenotype shared by all Black adults. When investigating dietary response, researchers should measure differences in insulin secretion, clearance, and tissue sensitivity directly, alongside factors such as adiposity, diet, and social and environmental exposures. The expression of these phenotypes is not simply a marker of greater insulin resistance; it reflects distinct kinetics of insulin secretion and extraction that lead to greater compensatory insulin demand and greater beta-cell strain under conditions of poor dietary quality. Although these findings point to biologically relevant differences, the mechanisms underlying such variability remain incompletely understood. Genetic polymorphisms [45], epigenetic modifications [46], and differences in gut microbiome composition and metabolic phenotypes [47] are all hypothesized contributors to the racial glucoregulatory differences. However, few controlled feeding studies or personalized nutrition trials have systematically evaluated how race moderates metabolic response to functional foods or nutrient-specific interventions, especially in young adult populations. This limitation highlights the importance of examining differential responses within metabolically diverse samples.

Dietary intervention outcomes may vary with baseline metabolic characteristics, habitual diet, food access, and other lived conditions. Studies should measure these factors directly and examine whether intervention outcomes differ across self-identified racial groups, without assuming that race itself determines biological response. Improving overall diet quality, particularly by increasing intake of fruits, vegetables, nuts, whole grains, and unsaturated fats, is a foundational strategy for reducing cardiometabolic risk across populations. Clinical trials with functional foods such as almonds, walnuts, and whole grains have shown improvements in fasting glucose, insulin sensitivity measures, and oral glucose tolerance test results, with effects observed in short-term studies and over several weeks of intervention [48–50]. However, the magnitude and sustainability of benefits from improving diet quality may differ across racial and ethnic groups due to differences in baseline dietary patterns, metabolic risk profiles, and structural determinants that shape food access and adherence. Interventions that fail to account for these contextual factors may yield uneven benefits and inadvertently perpetuate health disparities [51]. Accordingly, evaluating how racially diverse young adults respond to structured improvements in diet quality is critical for informing equitable dietary strategies and optimizing cardiometabolic health outcomes.

It is important to note that substantial heterogeneity exists within racial groups, and many observed differences may attenuate after accounting for diet quality, socioeconomic status, and environmental exposures. Thus, race should not be interpreted as a deterministic biological category but rather as a proxy for the interaction of biological and structural factors.

## Diet Quality, Functional Foods, and Structural Access

### The Evidence Gap in Dietary Pattern Research

The evidence supporting high-quality dietary patterns and their cardiometabolic benefits is strong. Adherence to the Mediterranean diet decreases the risk of cardiovascular events by about 30% in the PREDIMED trial [3], and following the DASH diet lowers systolic blood pressure across all levels of sodium intake [4]. Likewise, higher diet quality indices are linked to 14–16% reductions in overall mortality in prospective cohort studies [26]. While these findings are scientifically significant, their application to race-centered precision nutrition has limitations. Most data were derived from older, predominantly White, European-origin populations, and these results have not been replicated, re-examined, or even studied in young Black adults with obesity, at the most risk for cardiometabolic issues.

Dietary quality assessment compounds the problem. Standard dietary indices such as Healthy Eating Index (HEI), Alternate Healthy Eating Index (aHEI), Alternate Mediterranean Diet Score (aMED), and Dietary Approaches to Stop Hypertension (DASH) were developed and validated in predominantly White reference populations. Specific scoring components, such as olive oil as primary fat, fish as primary protein, and wine consumption, are calibrated to Mediterranean culinary traditions. Applied to Black participants, these indices may systematically underestimate dietary quality and miscategorize culturally specific food practices as nutritionally deficient, reinforcing narratives of Black dietary inadequacy that are partly artefactual. Therefore, there is a need to expand such dietary index algorithms to adequately capture diverse food components that accurately represent the racial diversity within the population.

### Functional Food Mechanisms Relevant to Black Adults’ Biology

Functional foods, including fruits, vegetables, nuts, and whole grains, provide cardiometabolic benefits through mechanisms that directly target the biological vulnerabilities of Black adults (Supplementary Table 1). Dietary fiber from all four categories promotes the production of short-chain fatty acids (SCFAs), butyrate, propionate, and acetate, by colonic bacteria. These metabolites activate FFAR2/FFAR3 receptors on enteroendocrine L-cells, which stimulates the secretion of GLP-1 and PYY [27]. This process reduces postprandial insulin demand in the beta-cell-stressed glucoregulatory phenotype common among Black adults [28]. MUFA and PUFA from nuts replace dietary saturated fats, decrease de novo ceramide synthesis from palmitoyl-CoA, alleviate PP2A-mediated Akt inhibition, and restore GLUT4 translocation, factors involved in ceramide-driven insulin resistance, which are known to be elevated at baseline in Black adults [52]. Polyphenols found in berries, dark vegetables, and nuts inhibit NF-κB-mediated production of pro-inflammatory cytokines in adipose tissue macrophages, reducing M1 polarization and subsequently decreasing IRS-1 serine phosphorylation [53, 54]. Furthermore, polyphenols selectively promote the growth of Akkermansia muciniphila, Lactobacillus, and Bifidobacterium in the gut microbiome [55]. Collectively, these findings point to race-specific benefits in functional food-based intervention.

Crucially, traditional African American dietary patterns contain several nutrient-dense foods that align with these metabolic mechanisms. Legumes such as black-eyed peas and kidney beans provide soluble fiber, resistant starch, and a low glycemic index; leafy greens, including collard and mustard greens, are rich in sulforaphane, folate, and calcium and activate Nrf2 pathways; sweet potatoes supply β-carotene, anthocyanins, and complex carbohydrates; and okra contributes mucilaginous soluble fiber that attenuates postprandial glycemic responses. Building on these existing dietary assets, culturally responsive functional food interventions that incorporate nuts, diverse fruits, and raw vegetables can translate key Mediterranean dietary principles into familiar food contexts. This approach avoids imposing culturally incongruent dietary patterns and may enhance adherence, acceptability, and long-term sustainability in Black communities.

### Structural Barriers to Functional Food Access

Dietary quality disparities in Black communities reflect structural conditions such as food deserts, economic constraints, and targeted marketing of ultra-processed foods, which are racially concentrated by historical design rather than individual preference. Food deserts are disproportionately located in Black neighborhoods, not because of poverty alone but because of decades of racially discriminatory retail disinvestment [56]. Functional food interventions that do not account for these structural conditions, that prescribe dietary changes without providing foods or addressing cost and availability barriers, will consistently underperform in Black communities regardless of their biological rationale. Race-stratified dietary intervention research must address this directly through study food provision, community-based recruitment infrastructure, and culturally concordant dietary counseling that situates recommendations within participants’ actual food environments.

## Race, Gut Microbiome, and Metabolomics

### Racial Differences in Baseline Gut Microbiome Composition

The gut microbiome is a central mediator of the relationship between dietary quality and cardiometabolic health. Studies have reported associations between self-identified race or ethnicity and gut microbiome composition, including differences detectable early in life [57]. Multiple cross-sectional and cohort studies document lower alpha- and beta-diversity, relative abundance of SCFA-producing taxa (such as *Faecalibacterium prausnitzii*,* Roseburia intestinalis*, Bifidobacterium), and higher abundance of pro-inflammatory taxa (such as *Bacteroides fragilis*,* Collinsella aerofaciens*) in Black compared with White adults [58]. The direction and size of these associations should be described within the populations studied rather than presented as a microbiome profile shared by Black or White adults. Diet, household and neighborhood conditions, medication exposure, and other life-course factors may contribute to observed patterns. Since much of this evidence is observational, these comparisons alone cannot establish the causes of microbiome differences or their effects on cardiometabolic risk and dietary response.

These differences partially reflect habitual dietary quality; predominantly, lower fiber intake that reduces the substrate available to SCFA-producing fermenters. In some studies, the differences persist after dietary adjustment, implicating early-life colonization conditions (lower breastfeeding rates, higher cesarean section rates), cumulative antibiotic exposure, and chronic psychosocial stress-driven dysregulation of the gut-brain axis as additional racial determinants of microbiome composition [59]. Together, these findings suggest that racial differences in gut microbiome composition arise from interacting dietary, environmental, and life-course exposures, with important implications for cardiometabolic risk and dietary responsiveness.

Microbial function may influence cardiometabolic regulation via four well-characterized pathways (Supplementary Table 2): (1) Lipopolysaccharide (LPS) translocation and metabolic endotoxemia: depletion of butyrate-producing taxa impairs colonocyte tight junction expression, increasing intestinal permeability and LPS translocation into systemic circulation. These events trigger TLR4 activation, which drives M1 macrophage polarization and IRS-1 serine phosphorylation, underpinning insulin resistance [60]. (2) SCFA deficiency: reduced abundance of butyrate-producing taxa such as Faecalibacterium and Roseburia decreases colonic short-chain fatty acid production, which in turn attenuates FFAR2/3-mediated GLP-1 secretion, reduces incretin signaling, and increases postprandial insulin demand [61]. (3) Trimethylamine N-oxide (TMAO) production: though the racial pattern of TMA lyase activity is incompletely characterized, altered gut microbiome composition modifies TMAO production from dietary choline and carnitine, contributing to atherosclerosis and platelet hyperreactivity [62]. (4) Bile acid transformation deficit: depletion of 7-alpha-dehydroxylating taxa reduces secondary bile acid production, attenuating FXR- and TGR5-mediated hepatic metabolic regulation and L cell GLP-1 stimulation [63]. This parallel incretin deficit compounds the impairment of the SCFA pathway. These mechanisms provide hypotheses for studying whether measured differences in microbial composition or function are associated with insulin-related outcomes. The available comparisons by self-identified race do not establish that these pathways operate differently in all Black and White adults, or that observed microbial differences cause disparities in cardiometabolic risk.

### The Greater Microbial Plasticity Hypothesis

The greater microbial plasticity hypothesis posits that individuals with lower baseline gut microbiome diversity and depletion of SCFA-producing taxa will exhibit larger compositional shifts in response to dietary intervention. This hypothesis predicts that participants with lower baseline abundance of fiber-fermenting taxa may show larger increases in those taxa after a fiber-rich intervention, regardless of self-identified race. Baseline diversity, habitual fiber intake, and microbial composition should be measured directly. Analyses can then assess whether intervention responses differ across racial groups and whether measured baseline factors help explain any observed differences. This prediction is grounded in ecological principles. In microbial communities where SCFA-producing taxa are depleted due to chronic substrate limitation, identical increases in dietary fiber can produce disproportionately larger expansions in these taxa because they are further from substrate saturation. Consistent with this “floor effect,” dietary fiber intervention studies demonstrate that the magnitude of microbial expansion is often inversely related to baseline abundance of fiber-fermenting taxa [64].

Despite its strong ecological and mechanistic basis, whether this greater plasticity extends across racial groups in response to functional food interventions remains untested. Addressing this question represents a critical, understudied opportunity to link baseline microbial ecology to differential cardiometabolic responses, with important implications for precision nutrition and health equity.

### Race-Specific Metabolomic Signatures and Dietary Response

Circulating metabolites reflect multiple influences, including diet, metabolic health, medication use, and environmental exposures. Cross-sectional metabolomic studies document elevated ceramide species (particularly Cer d18:1/16:0), elevated branched-chain amino acids (BCAAs: leucine, isoleucine, valine), elevated medium- and long-chain acylcarnitines, depleted antioxidant metabolites (glutathione, tocopherols), and elevated kynurenine/tryptophan ratios in Black adults relative to White adults at comparable adiposity [65, 66]. Each of these signatures reflects a specific mechanistic pathway relevant to insulin resistance. These include the ceramide-PP2A-Akt-GLUT4 impairment; BCAA-mTORC1-S6K1-IRS-1 serine phosphorylation; acylcarnitine-evidenced incomplete mitochondrial fatty acid oxidation; and inflammatory IDO1 activation of the kynurenine pathway. The latter connects allostatic load from chronic racial discrimination to a metabolomically measurable biochemical endpoint. The consistency of these findings across populations and their underlying causes require further evaluation. Although the metabolites are linked to pathways relevant to insulin resistance, a group-level association does not establish pathway activity in an individual or predict their response to a dietary intervention. Longitudinal studies should measure baseline metabolites and relevant exposures directly when testing whether they predict response (Supplementary Table 3).

Diet-induced metabolomic improvements following Mediterranean-style dietary patterns are well characterized in predominantly White populations. These improvements include reductions in ceramides, acylcarnitine, increases in PUFA-derived anti-inflammatory lipid mediators, and enhanced TCA cycle flux [67, 68]. Whether baseline ceramide and acylcarnitine concentrations predict the magnitude of change after dietary intervention remains to be tested in diverse populations. Similarly, whether FADS1/FADS2 genotype modifies the response to increased dietary ALA should be evaluated using measured genotype, dietary intake, and circulating fatty acid concentrations. Self-identified race can be examined in analyses of representation and differences in outcomes, but it cannot establish baseline metabolite concentrations, genotype, or an individual’s capacity to convert ALA to EPA.

## Toward Race-Centered Precision Nutrition

### The Cumulative Racial Biological Phenotype Framework

The evidence reviewed here supports a framework in which cardiometabolic risk reflects interactions among structural and environmental exposures, habitual diet, and individual biological characteristics that differ by race. These characteristics may include genetic variants, insulin secretion and clearance, adiposity, gut microbiome composition, and circulating metabolites. Self-identified race can help identify inequities in exposures and outcomes, but it does not establish an individual’s ancestry, biological profile, or likely response to a diet. Each proposed factor should therefore be measured directly.

The framework proposes that these factors may interact. For example, genetic variation could modify a response to dietary fatty acids, while habitual fiber intake could influence microbial composition and short-chain fatty acid production. Such relationships offer testable hypotheses; the evidence reviewed here does not establish that the pathways act together, amplify one another, or produce a single phenotype shared by Black adults.

A dietary intervention that increases fiber-rich foods, unsaturated fats, and micronutrient intake may affect several cardiometabolic pathways. The magnitude of benefit may depend on participants’ measured baseline characteristics, dietary intake, and ability to obtain and maintain the recommended foods. Future trials should test these potential modifiers directly and examine whether intervention access or outcomes differ across self-identified racial groups.

### The Greater Plasticity Hypothesis: Predictions and Falsifiability

The greater plasticity hypothesis proposed here posits that individuals with greater baseline metabolic perturbation, characterized by lower diet quality, altered gut microbiome composition, and metabolomic dysregulation, may exhibit larger absolute improvements in response to high-quality dietary interventions. The greater plasticity hypothesis predicts that participants with specified baseline characteristics, such as lower dietary fiber intake or higher concentrations of selected cardiometabolic risk markers, may show larger changes in those measures after dietary improvement. Structural barriers could also affect intervention uptake and sustained adherence and should be examined separately. Whether these characteristics or responses differ across self-identified racial groups is an empirical question.

This hypothesis generates specific, testable predictions across biological domains. On the glucoregulatory axis: a larger absolute increase in insulin sensitivity index (race-by-treatment interaction, *p* < 0.05) and a larger reduction in postprandial insulin in Black versus White intervention participants. On the gut microbiome axis: larger absolute alpha-diversity gains, greater enrichment of Faecalibacterium and Roseburia, larger fecal SCFA concentration increases, and stronger microbiome-insulin sensitivity mediation in Black participants. On the metabolomic axis: larger reductions in ceramide species, BCAAs, and acylcarnitines; larger increases in anti-inflammatory PUFA lipid mediators; and stronger ceramide-Akt and SCFA-GLP-1 mediation of insulin sensitivity improvement in Black adults. Cross-domain coherence, positive correlations between microbiome diversity gains, metabolomic improvement magnitude, and insulin sensitivity index improvement within Black participants, provide the most rigorous test of the integrated hypothesis.

### Requirements for Equitable Precision Nutrition Science

Closing the racial evidence gap in cardiometabolic nutrition science requires four design commitments that represent genuine departures from current field norms. First, race as a primary variable: studies powered for race-by-treatment interaction effects (requiring sample sizes 3–4× larger than overall-effect-powered trials), with pre-specified race-stratified analyses and formal interaction testing, not post hoc subgroup examination. Second, structural and cultural engagement: study food provision to remove food access barriers, community-based recruitment infrastructure, culturally concordant research teams, and personalized dietary counseling that situates functional food recommendations within participants’ existing food traditions rather than prescribing culturally alien dietary identities. Third, multi-omics integration: simultaneous OGTT-based glucoregulatory assessment, shotgun metagenomic gut microbiome profiling with fecal SCFA measurement, and metabolomics using different platforms in the same participants at the same time points, enabling the within-person mediation analyses that require co-variation data that separate studies cannot provide. Fourth, algorithmic equity: ensuring that precision nutrition prediction models, metabolomic response algorithms, polygenic risk scores, microbiome-guided dietary recommendations, are trained and validated on racially diverse datasets rather than predominantly European-ancestry cohorts, preventing the equity trap in which sophisticated personalized tools systematically underperform for the populations at greatest need.

## Knowledge Gaps, Research Priorities, and Conclusions

### Four Critical Knowledge Gaps

Four interlinked knowledge gaps prevent the generation of race-specific precision nutrition recommendations. Gap 1: No race-stratified glucoregulatory dietary intervention trial in young adults. Gap 2: No characterization of race-specific gut microbiome compositional and functional response to dietary improvement in young adults. Gap 3: No multi-platform metabolomic study of functional food dietary response in young Black versus White adults, including lipidomic ceramide/acylcarnitine profiling and kynurenine pathway biogenic amine assessment. Gap 4: No integrated multi-omics dietary intervention in racially diverse young adults capable of testing microbiome-metabolome-glucoregulation mediation pathways by race. These gaps cannot be closed sequentially: the mediation questions are within-person and require simultaneous multi-omics data collection from the same racially diverse participants. They must be addressed simultaneously in a single integrative study, with race as the primary analytical variable.

### Conclusions

This review has synthesized evidence from epidemiology, genetics, glucoregulatory physiology, epigenetics, food systems research, gut microbial ecology, and metabolomics to establish a mechanistically comprehensive and conceptually unified case for race-centered cardiometabolic nutrition research in young adults. The cumulative racial biological phenotype framework integrates the biological, structural, and epigenetic dimensions of racial cardiometabolic risk into a model. The central prediction of the greater plasticity hypothesis is both theoretically grounded and empirically falsifiable. Young Black adults remain insufficiently represented in dietary intervention research relative to the cardiometabolic disparities documented in US population data. Greater inclusion is needed to determine whether interventions are accessible and effective across populations and whether measured baseline characteristics predict differences in response. The current evidence does not establish that Black adults, as a group, have greater biological capacity to benefit from dietary improvement.

Cardiometabolic nutrition research should include populations historically underrepresented in intervention studies and assess whether recommendations are feasible and effective across different social and environmental contexts. Trials should report outcomes by self-identified race when appropriate for evaluating equity, while directly measuring the dietary, structural, and biological factors proposed to influence response. Evidence drawn largely from predominantly White study populations should be evaluated for its applicability to other populations rather than assumed to generalize without testing. 

## Key References

- Shi S, Huang H, Huang Y, Zhong VW, Feng N. Lifestyle Behaviors and Cardiometabolic Diseases by Race and Ethnicity and Social Risk Factors Among US Young Adults, 2011 to 2018. J Am Heart Assoc Cardiovasc Cerebrovasc Dis. 2023;12:e028926. https://doi.org/10.1161/JAHA.122.028926
  - ○ This large NHANES analysis provides the most comprehensive and contemporaneous documentation of race-specific lifestyle risk factor clustering in young adults, establishing that young Black adults have the highest prevalence of poor dietary quality, inadequate physical activity, and inappropriate sleep duration of any racial group - the epidemiological foundation for the intervention priority argued throughout this review.
- Mallott EK, Sitarik AR, Leve LD, Cioffi C, Camargo CA, Hasegawa K, et al. Human microbiome variation associated with race and ethnicity emerges as early as 3 months of age. PLOS Biol. 2023;21:e3002230. https://doi.org/10.1371/journal.pbio.3002230
  - ○ This study establishes that racial differences in the gut microbiome emerge as early as 3 months of age and persist. This lays the foundation for the cumulative racial biological phenotype framework that drives the responses to dietary intervention presented in this review.
- Wu Y, Ehlert B, Metwally AA, Perelman D, Park H, Brooks AW, et al. Individual variations in glycemic responses to carbohydrates and underlying metabolic physiology. Nat Med. Nature Publishing Group; 2025;31:2232–43. https://doi.org/10.1038/s41591-025-03719-2
  - ○ This landmark multi-omics study demonstrated that postprandial glycemic responses to identical carbohydrate meals vary substantially between individuals and are linked to differences in insulin resistance, beta-cell function, gut microbiome composition, and metabolomic profiles. The findings provide mechanistic support for precision nutrition approaches and reinforce the hypothesis that distinct glucoregulatory phenotypes may produce race-specific metabolic responses to functional food interventions.

## Supplementary Information

Below is the link to the electronic supplementary material.

Supplementary Material 1

## Data Availability

No datasets were generated or analysed during the current study.

## Abbreviations

CMD: Cardiometabolic diseases
T2D: Type 2 diabetes
NHANES: national health and nutrition examination survey
DASH: dietary approaches to stop hypertension
MetS: metabolic syndrome
BMI: body mass index
MHO: metabolically healthy obesity
MUFA: monounsaturated fatty acids
PUFA: polyunsaturated fatty acids
LA: linoleic acid
AA: arachidonic acid
ALA: alpha-linolenic acid
EPA: eicosapentaenoic acid
DHA: docosahexaenoic acid
HDAC: histone deacetylase
HPA: hypothalamic-pituitary-adrenal
HEI: healthy eating index
aHEI: alternate healthy eating index
aMED: alternate mediterranean diet score
SCFA: short-chain fatty acids
LDL: C-low-density lipoprotein cholesterol
TG: triglycerides
AUC: area under the curve
LPS: lipopolysaccharide
TMAO: trimethylamine n-oxide
BCAA: branched-chain amino acids
