# Supplementary material for: Race, Diet, and Cardiometabolic Risk in US Young Adults: A Biosocial Framework Integrating Biological Mechanisms and Structural Inequities Toward Precision Nutrition
Source: Curr Nutr Rep. 2026 Oct 12;15(1):82. doi: 10.1007/s13668-026-00804-x (PMC13658382; doi:10.1007/s13668-026-00804-x)
Supplement: Supplementary file 1 [file 13668_2026_804_MOESM1_ESM.docx]

**Supplementary Fig. 1**

Obesity-Lifestyle interactions are risk factors for cardiometabolic diseases

**Supplementary Fig. 2**

Conceptual framework of the progressive destabilization of metabolically healthy obesity through lifespan

**Supplementary Fig. 3**

Conceptual pathway of metabolic dysfunction in obesity from adipocyte expansion to insulin resistance and inflammation

**Supplementary Table 1 Key functional food categories, primary bioactive components, and cardiometabolic mechanisms relevant to reducing cardiometabolic risks among Black adults.**

| **Food category** | **Key bioactives** | **Primary cardiometabolic mechanisms** | **Race-specific mechanistic relevance** | **Reference** |
| --- | --- | --- | --- | --- |
| **Nuts (almonds, walnuts)** | MUFA, PUFA (ALA), fiber, phytosterols, polyphenols, Mg | ↓ LDL-C; ↓ TG; ↑ insulin sensitivity; ↑ SCFA-producing taxa; ↓ B. fragilis; ↓ postprandial glycemia | ALA → EPA via high-activity FADS2 in Black adults; MUFA supports CD36-impaired fatty acid oxidation; gut diversity gain larger from lower baseline | [1–3] |
| **Fruits (berries, citrus, prunes, stone fruits)** | Anthocyanins, flavonoids, phenolic acids, pectin, vitamin C, carotenoids | NF-κB inhibition; AMPK activation; ↑ Akkermansia; ↓ oxidative stress; ↓ postprandial glucose; ↓ blood pressure | Anti-inflammatory mechanisms target adipose macrophage M1 polarization in Black adults; polyphenol-driven Akkermansia gain larger from lower baseline | [4] |
| **Vegetables (leafy greens, cruciferous, peppers, carrots)** | Sulforaphane, lutein, nitrates, folate, K, Mg, insoluble fiber, polyphenols | ↓ blood pressure (nitrates → NO pathway); ↑ Nrf2 antioxidant defense; ↑ gut microbial diversity; ↓ CRP; ↓ homocysteine | Nitrate → NO blood pressure lowering particularly relevant to high-prevalence salt-sensitive hypertension in Black adults; folate supports methyl donor pool for epigenetic repair | [5] |
| **Whole grains (oats, brown rice, whole wheat)** | Beta-glucan, arabinoxylan, resistant starch, B vitamins, Mg, phytate, tocotrienols | ↓ postprandial glucose and insulin AUC; ↑ SCFA production; ↑ satiety hormones (GLP-1, PYY); ↓ LDL-C; ↑ microbial substrate diversity | Postprandial insulin AUC reduction is especially impactful in beta-cell-stressed glucoregulatory phenotype of Black adults; diverse fiber fractions support broader microbiome diversity gains | [6,7] |

ALA = alpha-linolenic acid; MUFA = monounsaturated fatty acid; PUFA = polyunsaturated fatty acid; LDL-C = low-density lipoprotein cholesterol; TG = triglycerides; AUC = area under the curve; SCFA = short-chain fatty acid; GLP-1 = glucagon-like peptide-1; PYY = peptide YY; NO = nitric oxide; CRP = C-reactive protein.

**Supplementary Table 2 Gut microbiome-mediated mechanistic pathways linking racial dysbiosis to cardiometabolic disease in Black adults**

| **Pathway** | **Microbial mechanism** | **Metabolite / effector** | **Cardiometabolic effect** | **Race-specific amplification** | **Reference** |
| --- | --- | --- | --- | --- | --- |
| **LPS translocation / metabolic endotoxemia** | ↑ Gram-negative Bacteroides, Collinsella; ↓ butyrate producers → ↓ tight junctions | Circulating LPS → TLR4 activation | ↑ adipose macrophage M1; ↑ TNF-α, IL-6; ↑ IRS-1 serine phosphorylation → insulin resistance | ↑ LPS-producing taxa + ↓ butyrate producers in Black adults synergistically amplify endotoxemia | [8]. |
| **TMAO production** | TMA lyase activity of Anaerococcus, Clostridium, Desulfovibrio on dietary choline/carnitine | TMA → hepatic FMO3 → TMAO | Foam cell formation; impaired reverse cholesterol transport; platelet hyperreactivity; vascular inflammation | Race-specific TMA lyase activity not yet characterized; dietary precursor profile differs by Black food culture | [9] |
| **SCFA deficiency → impaired incretin signaling** | ↓Faecalibacterium, Roseburia, Eubacterium → ↓ butyrate and propionate production | ↓ SCFA → ↓ FFAR2/3 → ↓ GLP-1, PYY secretion | ↑ postprandial glucose; ↑ compensatory insulin demand; ↑ hepatic glucose output; progressive beta-cell strain | Amplified by beta-cell hyperresponsive glucoregulatory phenotype in Black adults; greater beta-cell strain from lower GLP-1 tone | [10] |
| **Bile acid transformation deficit** | ↓ 7α-dehydroxylating Clostridium XIVa, Lachnospiraceae → altered secondary bile acid pool | ↓ DCA, LCA → ↓ FXR and TGR5 agonism | ↓ hepatic lipid clearance; ↓ TGR5-mediated GLP-1 secretion; ↑ hepatic gluconeogenesis | Additive with SCFA-GLP-1 deficit to produce substantially lower incretin tone in Black adults | [11] |

LPS = lipopolysaccharide; TLR4 = toll-like receptor 4; TMA = trimethylamine; TMAO = trimethylamine N-oxide; FMO3 = flavin-containing monooxygenase 3; SCFA = short-chain fatty acid; FFAR = free fatty acid receptor; GLP-1 = glucagon-like peptide-1; PYY = peptide YY; FXR = farnesoid X receptor; TGR5 = Takeda G protein-coupled receptor 5; DCA = deoxycholic acid; LCA = lithocholic acid.

**Supplementary Table 3 Race-specific baseline metabolomic signatures in Black versus White adults: biochemical classes, specific markers, mechanistic links to cardiometabolic disease, and predicted dietary intervention responses**

| **Metabolomic class** | **Key markers elevated in Black adults** | **Mechanistic link to CMD** | **Predicted dietary response direction** | **Reference** |
| --- | --- | --- | --- | --- |
| **Sphingolipids / ceramides** | Cer d18:1/16:0, sphingomyelin species, dihydroceramides | PP2A activation → Akt inhibition → ↓ GLUT4 translocation; beta-cell lipotoxicity and apoptosis | ↓ ceramide species via ↓ palmitate, ↑ MUFA/PUFA; greater reduction predicted in Black adults with higher baseline | [12] |
| **Branched-chain amino acids (BCAAs)** | Leucine, isoleucine, valine, BCKAs (2-oxoisovalerate, 2-oxoisocaproate) | mTORC1/S6K1 → IRS-1 serine phosphorylation → insulin resistance; BCKDH inhibition self-reinforcing cycle | ↓ BCAA via ↑ gut microbial BCAA catabolism; ↑ BCAA-fermenting taxa with dietary fiber | [13] |
| **TCA cycle intermediates** | Succinate, fumarate; altered citrate:succinate ratios | Succinate → SUCNR1 → macrophage M1 polarization, vascular inflammation; impaired mitochondrial flux | ↓ succinate, ↑ TCA flux markers via improved mitochondrial metabolic flexibility | [14,15] |
| **Oxidative**  **stress markers** | 8-isoprostane, MDA, 4-HNE; ↓ GSH, tocopherols | Lipid peroxidation → endothelial dysfunction; ↓ antioxidant reserves → amplified ROS-mediated inflammatory signaling | ↑ GSH, tocopherols via nut/fruit polyphenol/Vit E; ↓ isoprostanes; larger gains expected from lower antioxidant baseline | [16] |
| **Acylcarnitines** | Medium-chain (C8–C12) and long-chain (C14–C18) acylcarnitines; C18:1-acylcarnitine | Incomplete FAO → acyl-CoA accumulation → ceramide synthesis amplification, PDH inhibition → glucose intolerance | ↓ acylcarnitines via ↑ AMPK/PGC-1α → mitochondrial biogenesis; ↓ malonyl-CoA → ↑ CPT1 activity | [17] |
| **Biogenic**  **amines / neurotransmitters** | Serotonin precursors, kynurenine pathway intermediates; altered catecholamine metabolites | Kynurenine/tryptophan ratio reflects IDO1 inflammatory activation; gut-brain axis modulation of appetite and stress response | ↓ kynurenine/tryptophan ratio via ↓ IDO1 activation following anti-inflammatory dietary change | [18] |

Cer = ceramide; BCAA = branched-chain amino acid; BCKA = branched-chain keto acid; TCA = tricarboxylic acid; MDA = malondialdehyde; 4-HNE = 4-hydroxynonenal; GSH = glutathione; FAO = fatty acid oxidation; PDH = pyruvate dehydrogenase; CPT1 = carnitine palmitoyltransferase 1; AMPK = AMP-activated protein kinase; PGC-1α = peroxisome proliferator-activated receptor gamma coactivator 1-alpha; IDO1 = indoleamine 2,3-dioxygenase 1.

**References**

1. Liu X, Guasch‐Ferré M, Drouin‐Chartier J, Tobias DK, Bhupathiraju SN, Rexrode KM, et al. Changes in Nut Consumption and Subsequent Cardiovascular Disease Risk Among US Men and Women: 3 Large Prospective Cohort Studies. J Am Heart Assoc. Wiley; 2020;9:e013877. https://doi.org/10.1161/JAHA.119.013877

2. Ukhanova M, Wang X, Baer DJ, Novotny JA, Fredborg M, Mai V. Effects of almond and pistachio consumption on gut microbiota composition in a randomised cross-over human feeding study. Br J Nutr. 2014;111:2146–52. https://doi.org/10.1017/S0007114514000385

3. Sergeant S, Hugenschmidt CE, Rudock ME, Ziegler JT, Ivester P, Ainsworth HC, et al. Differences in arachidonic acid levels and fatty acid desaturase (FADS) gene variants in African Americans and European Americans with diabetes or the metabolic syndrome. Br J Nutr. 2012;107:547–55. https://doi.org/10.1017/S0007114511003230

4. Belwal T, Nabavi SF, Nabavi SM, Habtemariam S. Dietary Anthocyanins and Insulin Resistance: When Food Becomes a Medicine. Nutrients [Internet]. Multidisciplinary Digital Publishing Institute; 2017 [cited 2026 Mar 21];9. https://doi.org/10.3390/nu9101111

5. Kapil V, Khambata RS, Robertson A, Caulfield MJ, Ahluwalia A. Dietary nitrate provides sustained blood pressure lowering in hypertensive patients: a randomized, phase 2, double-blind, placebo-controlled study. Hypertension. Dallas, Tex. : 1979; 2015;65:320–7. https://doi.org/10.1161/HYPERTENSIONAHA.114.04675

6. Koh A, De Vadder F, Kovatcheva-Datchary P, Bäckhed F. From Dietary Fiber to Host Physiology: Short-Chain Fatty Acids as Key Bacterial Metabolites. Cell. 2016;165:1332–45. https://doi.org/10.1016/j.cell.2016.05.041

7. Whitehead A, Beck EJ, Tosh S, Wolever TM. Cholesterol-lowering effects of oat β-glucan: a meta-analysis of randomized controlled trials1234. Am J Clin Nutr. 2014;100:1413–21. https://doi.org/10.3945/ajcn.114.086108

8. Cani PD, Amar J, Iglesias MA, Poggi M, Knauf C, Bastelica D, et al. Metabolic Endotoxemia Initiates Obesity and Insulin Resistance. Diabetes. 2007;56:1761–72. https://doi.org/10.2337/db06-1491

9. Formation of propionate and butyrate by the human colonic microbiota - Louis - 2017 - Environmental Microbiology - Wiley Online Library [Internet]. [cited 2026 Mar 21]. https://enviromicro-journals.onlinelibrary.wiley.com/doi/full/10.1111/1462-2920.13589?utm_source=chatgpt.com. Accessed 21 Mar 2026

10. Qi J, You T, Li J, Pan T, Xiang L, Han Y, et al. Circulating trimethylamine N-oxide and the risk of cardiovascular diseases: a systematic review and meta-analysis of 11 prospective cohort studies. J Cell Mol Med. 2018;22:185–94. https://doi.org/10.1111/jcmm.13307

11. Thomas C, Gioiello A, Noriega L, Strehle A, Oury J, Rizzo G, et al. TGR5-mediated bile acid sensing controls glucose homeostasis. Cell Metab. 2009;10:167–77. https://doi.org/10.1016/j.cmet.2009.08.001

12. Summers SA. Ceramides in insulin resistance and lipotoxicity. Prog Lipid Res. 2006;45:42–72. https://doi.org/10.1016/j.plipres.2005.11.002

13. Newgard CB, An J, Bain JR, Muehlbauer MJ, Stevens RD, Lien LF, et al. A Branched-Chain Amino Acid-Related Metabolic Signature that Differentiates Obese and Lean Humans and Contributes to Insulin Resistance. Cell Metab. Elsevier; 2009;9:311–26. https://doi.org/10.1016/j.cmet.2009.02.002

14. Tannahill GM, Curtis AM, Adamik J, Palsson-McDermott EM, McGettrick AF, Goel G, et al. Succinate is an inflammatory signal that induces IL-1β through HIF-1α. Nature. Nature Publishing Group; 2013;496:238–42. https://doi.org/10.1038/nature11986

15. Keiran N, Ceperuelo-Mallafré V, Calvo E, Hernández-Alvarez MI, Ejarque M, Núñez-Roa C, et al. SUCNR1 controls an anti-inflammatory program in macrophages to regulate the metabolic response to obesity. Nat Immunol. 2019;20:581–92. https://doi.org/10.1038/s41590-019-0372-7

16. Billingsley HE, Carbone S. The antioxidant potential of the Mediterranean diet in patients at high cardiovascular risk: an in-depth review of the PREDIMED. Nutr Diabetes. 2018;8:13. https://doi.org/10.1038/s41387-018-0025-1

17. Adams SH, Hoppel CL, Lok KH, Zhao L, Wong SW, Minkler PE, et al. Plasma Acylcarnitine Profiles Suggest Incomplete Long-Chain Fatty Acid β-Oxidation and Altered Tricarboxylic Acid Cycle Activity in Type 2 Diabetic African-American Women. J Nutr. 2009;139:1073–81. https://doi.org/10.3945/jn.108.103754

18. Badawy AA-B. Kynurenine Pathway of Tryptophan Metabolism: Regulatory and Functional Aspects. Int J Tryptophan Res. SAGE Publications Ltd STM; 2017;10:1178646917691938. https://doi.org/10.1177/1178646917691938
